# LeishMANIAdb: a comparative resource for *Leishmania* proteins

**DOI:** 10.1093/database/baad074

**Published:** 2023-10-31

**Authors:** Gábor E Tusnády, András Zeke, Zsófia E Kálmán, Marie Fatoux, Sylvie Ricard-Blum, Toby J Gibson, Laszlo Dobson

**Affiliations:** Protein Bioinformatics Research Group, Institute of Enzymology, Research Centre for Natural Sciences, Magyar Tudósok körútja 2, Budapest 1117, Hungary; Department of Bioinformatics, Semmelweis University, Tűzoltó u. 7, Budapest 1094, Hungary; Protein Bioinformatics Research Group, Institute of Enzymology, Research Centre for Natural Sciences, Magyar Tudósok körútja 2, Budapest 1117, Hungary; Faculty of Information Technology and Bionics, Pázmány Péter Catholic University, Práter u. 50/A, Budapest 1083, Hungary; ICBMS UMR CNRS 5246, University Lyon 1, Rue Victor Grignard, Villeurbanne 69622, France; UMR CNRS 5086, University Lyon 1, 7 Passage du Vercors, Lyon 69367, France; ICBMS UMR CNRS 5246, University Lyon 1, Rue Victor Grignard, Villeurbanne 69622, France; Structural and Computational Biology Unit, European Molecular Biology Laboratory, Meyerhofstraße 1, Heidelberg 69117, Germany; Protein Bioinformatics Research Group, Institute of Enzymology, Research Centre for Natural Sciences, Magyar Tudósok körútja 2, Budapest 1117, Hungary; Structural and Computational Biology Unit, European Molecular Biology Laboratory, Meyerhofstraße 1, Heidelberg 69117, Germany

## Abstract

Leishmaniasis is a detrimental disease causing serious changes in quality of life and some forms can lead to death. The disease is spread by the parasite *Leishmania* transmitted by sandfly vectors and their primary hosts are vertebrates including humans. The pathogen penetrates host cells and secretes proteins (the secretome) to repurpose cells for pathogen growth and to alter cell signaling via host–pathogen protein–protein interactions). Here, we present LeishMANIAdb, a database specifically designed to investigate how *Leishmania* virulence factors may interfere with host proteins. Since the secretomes of different *Leishmania* species are only partially characterized, we collated various experimental evidence and used computational predictions to identify *Leishmania* secreted proteins to generate a user-friendly unified web resource allowing users to access all information available on experimental and predicted secretomes. In addition, we manually annotated host–pathogen interactions of 211 proteins and the localization/function of 3764 transmembrane (TM) proteins of different *Leishmania* species. We also enriched all proteins with automatic structural and functional predictions that can provide new insights in the molecular mechanisms of infection. Our database may provide novel insights into *Leishmania* host–pathogen interactions and help to identify new therapeutic targets for this neglected disease.

**Database URL:**  https://leishmaniadb.ttk.hu/

## Introduction

Leishmaniasis is a neglected tropical disease causing severe symptoms, affecting around 1 million new people yearly, with annual deaths estimated to be around 60 000 (1). Although over 90% of cases occur in poor regions south of the Equator, due to climatic changes it also appears in new areas, and it has already shown up in Mediterranean European countries (2) and Texas, USA (3). To this date, no approved human vaccine is available and treatment is most effective at an early stage of the infection. *Leishmania* parasites are unicellular, flagellated trypanosomatids, belonging to the class Kinetoplastea. Upon infection, the amastigote stage pathogen (with reduced flagella) is engulfed by phagocytes, where it ends up in a stable parasitophorous vacuole that protects it (4). *Leishmania* cells then proliferate unhindered within host cells until egress and spreading to nearby phagocytes (5). The parasite secretes proteins that enter various parts of the cell (6). The secreted virulence factors can then interfere with cell signaling by interacting with the host proteins: they increase glycolytic metabolism (7), perturb microbicidal pathways (8), escape the innate immune response, and repurpose macrophages for parasite replication (9) by disturbing cellular protein–protein interactions (PPIs). Interestingly, these mechanisms are somewhat unique to *Leishmania* among trypanosomes, which are mostly extracellular pathogens and do not enter host cells. In contrast, *Leishmania* secretes proteins which are critical for host cell subjugation, but how they enter the cytoplasm of host cells is still poorly understood.

In many distant, unrelated intracellular pathogens, ranging from viruses and bacteria to unicellular eukaryotes, the host targeted interactions are often mediated via Short Linear Motifs (SLiMs) (10). SLiMs are flexible protein segments composed of a restricted number of residues (between 3 and 10) that usually bind to structured protein domains. Their short length and structural flexibility enable them to bind to a wide range of domains. Cellular SLiMs typically bind their targets with low micromolar affinity. These weak and transient interactions enable SLiMs to work in cooperative regulatory systems (11). Pathogens mimic host SLiMs to interact with host cell proteins (10). Pathogen SLiMs often bind with higher affinities than the cellular ones, outcompeting the native interactions, permanently re-wiring the host regulation network. A few modulatory SLiMs have already been discovered in eukaryotic pathogens, such as the *Toxoplasma gondii* MapK docking motif (12) and the stage-specific (promastigote–amastigote) phosphorylation motifs from *Leishmania* (13). In addition, several putative SLiMs were recently detected in *Leishmania*, such as heparin-binding sequences or RGD integrin-binding motifs though their function have not yet been confirmed yet (14).

Numerous studies investigated *Leishmania* secretomes. Most of them expose promastigotes to a heat shock and pH change (attempting to emulate the conditions that promote promastigote-to-amastigote stage transition) and then analyze the *Leishmania* conditioned medium by proteomics to identify secreted proteins (15), and measure their protein abundance or by transcriptomics to detect mRNA levels (16). While high-throughput experiments inherently suffer from a certain level of noise, experiments on individual proteins may be more reliable—in the case of *Leishmania* the vast majority focuses on leishmanolysin (GP63), a surface-anchored protease important for pathogenesis (17, 18). Furthermore, data were collected on different *Leishmania* species/strains identified via names and identifiers varying from one source to another, making a unified overview challenging. Another key step toward understanding the infection mechanism would be the identification of *Leishmania* surface proteins that can mediate the attachment of the pathogen to the host cell. Some surfaceome experiments were carried out on *Leishmania*-related species, and human host proteins binding to the surface of 24 strains of intact *Leishmania* have been identified (19). Besides the characterization of *Leishmania* secretomes, the identification of host**–***Leishmania* PPIs is needed to narrow down virulence factors perturbing the host cell regulation to modules interfering with host proteins. SLiMs have low information content and simply scanning for matches to them in *Leishmania* secretomes may yield many false positives. Their structural and functional context, such as accessibility, conservation and localization, are all key elements to successfully identify those that may have a role in rewiring the host cell regulation. Notably, SLiMs also play a key role in maintaining housekeeping processes in *Leishmania*. Therefore, to find candidate SLiMs that may alter the host regulation, we need to discriminate SLiMs of proteins that reach the host cytoplasm or nucleus but limited information about these proteins are available. Currently, the only publicly available database dealing with *Leishmania* proteins is TriTrypDB (20), which is part of the VEuPathDB (21). TriTrypDB is a functional genomic resource for Trypanosomatidae, offering proteomic datasets; however, it does not focus on protein structure, protein motif search and interactions.

We developed LeishMANIAdb to expedite *Leishmania* research by unifying scattered information from the literature in a user-friendly way and to extend available resources by adding protein level information. We collected high-throughput experiments and interaction studies on individual proteins and used various prediction methods to enrich proteins with structural information.

## Results

### Selection of *Leishmania* proteomes and homology mapping of various kinetoplastid proteins

We selected five *Leishmania* species (reference proteomes: *L. brazliensis, L. donovani, L. infantum, L. major* and *L. mexicana*), thirteen *Leishmania* strains (*LbraziliensisMHOMBR75M2903, LbraziliensisMHOMBR75M2904, LbraziliensisMHOMBR75M2904_2019, LdonovaniBPK-282A1, LdonovaniCL-SL, LdonovaniHU3, LdonovaniLV9, LinfantumJPCM5, LmajorFriedlin, LmajorFriedlin2021, LmajorLV39c5, LmajorSD75.1 and LmexicanaMHOMGT2001U1103*) and six related species (reference proteomes: *Bodo saltans, Leptomonas seymouri, Trypansoma brucei, Trypansoma cruzi, Trypansoma rangeli and Trypanosoma theileri*) as an outgroup (22). *Leishmania* proteins were also cross-referenced to TriTrypDB (20). Around 30% of the cross-referenced proteins have different sequences deposited into these resources, and in most cases, the difference is due to the predicted position of the initiator methionine. For data compatibility, we always use the UniProt sequence version, but the conflicts are highlighted in LeishMANIAdb. When selecting the species we looked for those that have at least two strains deposited into TriTrypDB, and where cross-references and strain information were present and could be assigned with the least errors (Supplementary Table 1). We also performed a similarity search between these proteins and linked close homologs (only close kinetoplastid hits, see Methods) so annotations and predictions can be easily compared between them. All manual annotations and experimental data from different sources were mapped to these proteins. The 13 *Leishmania* strain proteomes were downloaded from TriTrypDB. Altogether LeishMANIAdb contains 40 537 searchable *Leishmania* proteins from reference proteomes, 108 766 proteins from different strains and 68 924 other kinetoplastid proteins to strengthen predictions.

### Manual annotation of host–pathogen PPIs and TM protein localization

We manually curated hundreds of proteins, using two strategies.

The first type of annotation was the collection of host–pathogen PPI experiments on individual proteins, with the majority of them involving leishmanolysin (GP63). We collected 29 papers reporting 82 *Leishmania* PPIs with different hosts. Although experiments were mapped back to specific proteins, the results are also displayed on close homologs (with a note that the experimental data is derived from a different protein) resulting in 211 proteins that contain PPI data. Interactions were reported using the Minimum Information required for reporting a Molecular Interaction eXperiment MIMIx (23) community standard description.

The second type of manually curated data was the localization and functional annotation of TM proteins. The aim was to find surface proteins that may facilitate the infection, but we annotated hundreds of other TM proteins with their localizations too. For this task, we used close homologous protein groups. Altogether 342 protein families were annotated and these annotations were shared between 3764 proteins (which is 45.11% percent of the predicted TM proteomes and 9.28% of all proteins of the 5 species combined).

### The definition of *Leishmania* secretome and protein localization is still incomplete

**Figure 1.**
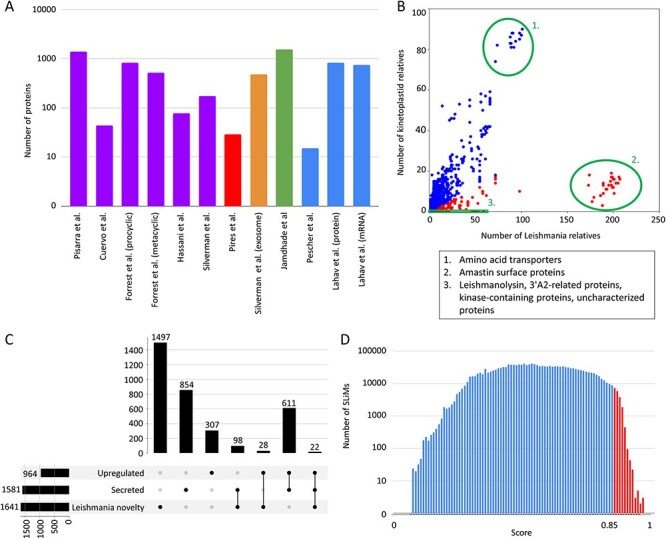
LeishMANIAdb content. All data were calculated on *Leishmania infantum*. A: Number of proteins in different proteomic datasets (purple: promastigote secretome, red: amastigote secretome, orange: exosome, green: housekeeping genes, blue: higher protein abundance level upon infection). B: Number of kinetoplastid and Leishmania close homologs. Each dot represents a protein (red: at least 80% of close homologs are in Leishmania, blue: other proteins). Green circles represent distinctive groups. C: Overlap between abundant, secreted and ‘Leishmania novelty’ proteins (for more detail see text). D: Distribution of all predicted SLiMs with different scores. Red marks candidate motifs above 0.85 cutoff (for more details see text).

*Leishmania* not only exploits host**–**secretory pathways to distribute effectors, but also utilizes an unusual mechanism to deliver proteins to the cytosol of infected cells by releasing exosomes into the parasitophorous vesicle, which might fuse with the vesicular membrane to release their protein content (24). Therefore, computational methods based on signal peptides and localization predictions are not sufficient to predict *Leishmania* secretomes. To overcome this limitation, we also used high-throughput experiments (15, 25–28) to increase the coverage of *Leishmania* secretomes. Strikingly, the number of proteins in these secretomes varies to a large extent. Other datasets include proteins found in glycosomes (29), stage-dependent (promastigote/amastigote) phosphoproteomics (13), housekeeping gene localizations (30), exosome content (24), protein and mRNA abundance data (16, 31). When we mapped back all secretome and abundance experiments to *Leishmania infantum* (from close homologous proteins of other *Leishmania* species), the number of identified proteins ranges from 10 to 2000 (Figure 1A), and even when experimental conditions were similar they yielded highly different amounts of proteins. For example, pioneer secretome studies only provided a few hundred hits, while the latest ones are more inclusive with thousands of hits.

Gene duplication is often acting on protein families responsible for host–pathogen PPIs; therefore, we also collected proteins that are highly expanded. Notably, as all kinetoplastids have a polycistronic transcription system, the main way to amplify expression of critical proteins is through gene duplication. Thereby, highly expanded gene families can be directly mapped to functions critical for these parasites (32). In this case, we could discriminate between proteins with many paralogs found in other kinetoplastids versus *Leishmania-*exclusive amplified proteins. When we searched for close homologs of *Leishmania infantum* proteins, we found distinct amino acid transporter and cofactor families already expanded in all kinetoplastids including *Leishmania*. In contrast, amastins, leishmanolysin, 3ʹA2-related proteins, kinase-containing putative receptor proteins (and several uncharacterized proteins) seemed to be highly abundant in *Leishmania* proteomes compared to all kinetoplastids (Figure 1B).

Comparing complete proteomic datasets yielded only a small overlap. We defined (1) *Leishmania*_novelty proteins, which are proteins without close homologs in SwissProt, without characterized Pfam domains, and expanded in *Leishmania infantum* (compared with other kinetoplastids); (2) abundant proteins, which are proteins showing increased abundance upon infection; and (3) secreted proteins experimentally identified in at least two secretome experiments. These definitions provided markedly different protein sets, with some overlap between secreted and abundant proteins (611 proteins) and with only 22 proteins contained in all datasets (Figure 1C).

### AlphaFold2 provides an alternative way estimate structural features

We used different methods to predict the structural features of proteins. Classical sequence-based methods can detect globular domains (33), TM regions (34) and intrinsically disordered regions (IDRs) (35). However, the use of AlphaFold2 (AF2) (36) provides alternative ways to obtain structural information. In LeishMANIAdb, we used structures available in the AlphaFold database (37) (however, we could not find 3192 proteins (∼6% of all *Leishmania* proteins in LeishMANIAdb)). We not only displayed the predicted 3D structure of the proteins, but also information derived from the AF2 models, such as the secondary structures and the position of the lipid bilayer for membrane proteins using the method introduced in the TmAlphaFold database (38). Although AF2 was originally built to predict protein structure, the scientific community quickly realized it is as much (if not more) efficient at predicting protein disorder (38). To analyze IDRs, we displayed predicted local distance difference test (pLDDT) values and relative surface accessibility from AF2. For IDR prediction in TM proteins, we tailored MemDis (39) to incorporate features from AF2 instead of sequence-based predictors (see Methods).

### Short linear motif candidates that may hijack host cell regulation

We scanned *Leishmania* proteins for SLiMs using the regular expressions stored in the Eukaryotic Linear Motif (ELM) resource (40). Scanning SLiMs alone would mostly yield false positive hits, so we developed a scoring system that ranges from 0 to 1, and that takes into account most information we collected. We aimed to develop a scoring system where conservation and accessibility/disorder has a reasonably high weight, while keeping in mind that proteomic experiments and localization information are a good way to narrow down the potentially large number of false positive hits. Unfortunately, due to the lack of data, in the case of *Leishmania*, it is not possible to construct a benchmark set to evaluate motif scores. We can still assume that a good starting point can be when most predictions and proteomic data agree. Considering *Leishmania infantum* alone, we detected over a million putative motifs, from which 1.21% had a score above 0.85, on 343 proteins (Figure 1D).

### The LeishMANIAdb web resource

**Figure 2.**
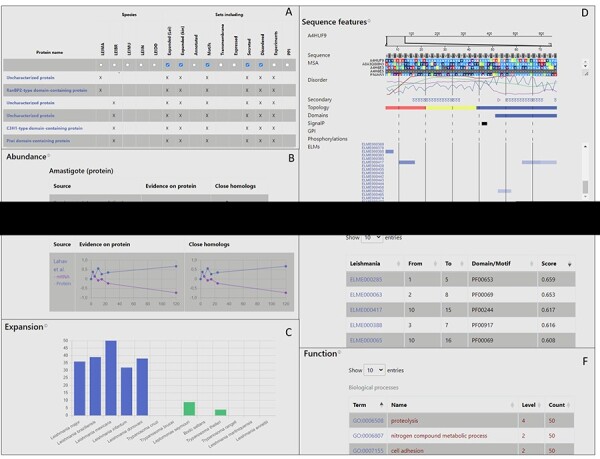
Layout of LeishMANIAdb. A: The search/browse result menu. B: Expression section of the entry page. C: Expansion section. D: Sequence features section. E: Putative motif mimicry section. F: Function section.

To visualize all the collected and calculated information, we developed an open-access resource. In LeishMANIAdb, users can search for proteins using their UniProt Accession (AC), Entry name (formerly ID), gene name and protein name. We also provide several protein sets as examples to help users browsing the database. Currently, proteins are sorted based on (1) species: *L. braziliensis, L. donovani, L. infantum, L. major* and *L. mexicana*; (2) manual curation data; (3) experimental data: secreted proteins, protein abundance/mRNA level data, proteins with any kind of experimental data listed above; (4) computationally predicted information: proteins expanded in *Leishmania* (score ≥ 0.8—see Methods, Supplementary Material), transmembrane (TM) proteins, proteins with high disordered content (at least 70% predicted disorder), proteins with high-scoring linear motifs (score ≥ 0.85), and novel kinetoplastid proteins (proteins without SwissProt homologs or Pfam domains). After searching (or selecting a protein set), users can further narrow their selection by choosing any other criterion (Figure 2A).

The entry page for each protein consists of up to 10 sections, which are only visible if they contain data. The ‘Quick info’ displays the protein name, species, cross-references, and its number of amino acid residues. Data curation appears under the ‘Annotations’ section. PPI (curated at the MIMIx level), localization, and function annotations are mirrored from close homologous proteins. We also display functional annotations for *Leishmania donovani* (and close homologs) by Jardim *et al.* (30) (last update in 2018). The ‘Localization’ section contains high-throughput experiment data—promastigote and amastigote secretion, an exosome experiment and the glycosome. Protein localization, signal peptide and glycosylphosphatidyl (GPI) anchor predictions are also displayed here. Since the reliability of both predictions and experiments may vary, we also display all this data for close homologous proteins, so users can quickly check the robustness of information—by checking if different sources on closely related proteins agree. Furthermore, we also collected Gene Ontology (GO) (41) annotations for cellular compartments. In this case, the specificity of the term (how deep it is on the tree) is shown in the level column. GO annotations are collected for all close homologous proteins too and the number of occurrences of each term is displayed. We highlighted terms that are associated with the inspected protein itself that is displayed on the page. The ‘Abundance’ module can display the mRNA and protein level experiments: static/single point (upregulated or not) or time-course experiments (e.g. mRNA and protein levels available for 7 timesteps across 120 h (16), Figure 2B). In the “Expansion” section, the number of close homologs are displayed by species with a color-code to identify *Leishmania* intracellular and extracellular/free-living relatives (Figure 2C). The ‘Sequence features’ displays various information (Figure 2D). At the top, the gapless multiple sequence alignment (MSA) of proteins from the reference proteomes is visible. In this alignment gaps from the entry protein were removed (the original alignment with all strains can be downloaded) so other protein features could be visualized. Protein disorder, secondary structures, TM topology prediction, domains, signal peptides and GPI anchors, and stage-dependent phosphorylation are also displayed. Predicted SLiMs are shown with a color-coded score (see Methods, Supplementary Material). In the ‘Structure’ section, the AF2 predicted structure is available (with the position of the membrane domain for TM proteins). The ‘Putative motif mimicry’ section is the table format version of SLiMs from the “Sequence features’’ module (Figure 2E). The ‘Function’ section contains GO Molecular Function and Biological Process terms. As done for the Cellular Component, terms are transferred from close homologous proteins, and they can be sorted based on their specificity (how deep they are located in the tree) and occurrences considering close homologs (Figure 2F). Finally, each result from a BLAST search against SwissProt and kinetoplastids relatives are listed in the ‘Homologs’ table (not only close homologs, see Methods).

For each protein the full MSA, high-throughput experiments, annotation and predicted sequential features can be downloaded from the bottom of the page. Batch download is also available to download the full database or different protein sets.

## Discussion

### Reliability of data

In LeishMANIAdb, we aimed to collect high-throughput experimental data, PPI data on individual proteins, predictions and localization information based on close homologs. We noticed that the amount of data from MS experiments differs highly, and therefore likely the quality also varies. Not unexpected from high-throughput techniques applied to less-studied organisms, this can be attributed to the quality of sample preparation, MS experimental techniques and most likely to the sequences in the background databases. One striking finding was that the secretory datasets contain a large number of proteins that are likely to take part in the housekeeping processes of *Leishmania* cells, such as cytoskeletal proteins, nuclear histones and metabolic enzymes. Exosomes are known to contain a relatively high amount of “background” proteins leaking from the cytosol of cells. Another explanation is that several housekeeping genes (such as intracellular chaperones and enzymes) are moonlighting proteins, they are generally constitutively expressed and have high levels of expression, while they are fulfilling other functions outside the cells (42). Due to the lack of comparative studies, we cannot assess the enrichment ratios of secreted molecules, to see if there is selective exosomal packaging of a well-defined subset of *Leishmania* proteins. However, *Leishmania* exosomal-like secretion also differs from the typical exosomal sorting seen in other eukaryotic organisms because budding primarily initiates at the cell membrane, and not inside multivesicular bodies (endosomes). Therefore, it is equally possible that in Leishmaniids, the budding is non-selective for its cytoplasmic cargos. Instead, it would be initiated by cell surface receptors and primarily serve as a defense mechanism against membrane-attached host complement and other immune complexes, removing them before they could damage the parasite membrane. Currently, testing of the latter hypothesis is impossible, since only soluble components, but not the integral membrane proteins of *Leishmania* exosomes have been studied in depth in the above cited studies.

**Figure 3.**
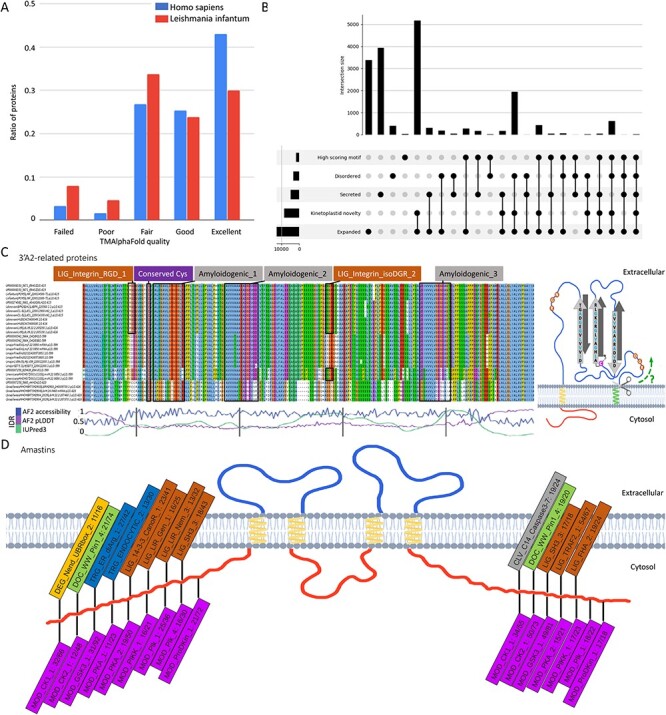
A: Distribution of membrane protein quality levels of AlphaFold structure in Homo sapiens and Leishmania infantum. B: Upset diagram of proteins that are (1) secreted, (2) novel kinetoplastid, (3) expanded (or new) in Leishmania, (4) disordered, (5) contain candidate SLiMs. C: left: Multiple Sequence Alignment of 3ʹA2 related proteins (alignment is available under UniProt AC: E9AGZ3). Amyloidogenic regions, conserved cysteine and integrin-binding motifs are highlighted; right: proposed topology of 3ʹA2 related proteins. D: Frequent SLiMs in the cytoplasmic tail regions of amastins (the numbers denote the unique/total occurrences).

From a computational point of view, predicting any features on *Leishmania* proteins might be highly challenging, as methods established were mostly trained on sequences that show little or no similarity to *Leishmania* proteins. The five *Leishmania* reference proteomes contain 10 267 uncharacterized proteins combined, which is ∼25% of LeishMANIAdb. TmAlphaFold provides an objective quality measurement option for α-helical membrane proteins. When we compared the TM proteome of *Homo sapiens* and *Leishmania infantum*, we noticed that the ratio of good and excellent quality structures was much lower in *Leishmania*, probably caused by the different coverage of kinetoplastid and human structures deposited into the PDB (Figure 3A).

### Case studies

LeishMANIAdb can be utilized for different purposes and can be a good starting point for various analyses. We selected three examples that highlight some use cases of the resource.

Using the Browse menu, after selecting a category, users can further narrow down their search for proteins selecting additional categories to refine the results. For instance, if users are looking for *Leishmania* SLiMs that may alter or rewire host cell regulation network, they can look for proteins that were experimentally proven to be secreted, and then select proteins with disordered regions because SLiMs are mostly located in IDRs. ‘Kinetoplastid novelty’ selection ensures that the protein and its domains are not present in organisms belonging to other lineages, while *Leishmania* novelty/expansions select proteins that are new or highly expanded in *Leishmania* species. Last, by selecting high-scoring motifs, users get a list of proteins where the motif is most likely to be functional (Figure 3B shows the Venn diagram of the selection). These proteins may be an interesting starting point for further analyses.

When performing systematic searches to identify possible parasite hits of integrin ligand motifs (that only function in the host, as kinetoplastids have no integrins), we identified a striking set of examples in a family of poorly-known *Leishmania* genes called 3ʹA2 related ORFs. This kinetoplastid-specific family of genes is actually expanded in *Leishmania* species together with the canonically secreted A2 proteins, which are known pathogenicity factors (43). While the actual sequences of these proteins are poorly conserved and very little is known about their subcellular location, the *Leishmania* versions have at least one TM region and a C-terminal cytoplasmic tail, with an N-terminal signal peptide (or possibly another TM segment). Nevertheless, in the predicted, largely disordered extracellular segment we observed multiple, short, conserved stretches that may have amyloidogenic properties (high Val, Ala and Gly content, upon visual inspection), presumably capable of oligomerization and amphiphilic interaction with membranes (Figure 3C). A highly conserved cysteine residue preceding the first amyloidogenic sequence might help the homodimerization by forming a disulfide bridge with neighboring 3ʹA2 related protein. Strikingly, in *Leishmania infantum* and *Leishmania donovani* (both species capable of causing visceral leishmaniasis), the N-terminus of these proteins carries canonical RGD (Arg-Gly-Asp) sequences, immediately after the putative signal peptide cleavage site. In addition, *Leishmania donovani* and *Leishmania infantum* proteins contain an NGR motif where asparagine deamidation might yield an isoDGR motif. If these proteins are expressed on the cell surface, they might bind to host integrins in an oligomeric state, and might even attack the host membrane as if it were a β-barrel pore-forming toxin. However, much more experiments are needed to test any of these hypotheses.

Amastins are a large family of kinetoplastid-specific membrane proteins that belong to the broader claudin-like superfamily, implicated in the maintenance of parasitophorous vacuoles (44). Accordingly, the majority of amastins have four tightly packed TM segments, with cytosolic tail regions. Similarly to their vertebrate counterparts that form tight cell–cell junctions by complex oligomerization processes, amastins might also engage in a variety of interactions with internal as well as external, host proteins (44). Although their exact function is not known, among the 221 identified amastins with 4 TM regions we looked for SLiMs that occur in multiple proteins. Predicted SLiMs (within disordered regions) were packed in their cytoplasmic tail regions (Figure 3D). Since these regions face inward the parasite, we further narrowed hits based on their binding domain to be present in *Leishmania*. We identified multiple potential phosphorylation sites and PPI motifs, such as SH3 ligands (*Leishmania* species do encode SH3 domain proteins) as well as vesicular trafficking signals. The tail region of amastins seems to be highly variable, likely acting as a hotspot in the pathogen–host arms race.

### Comparison with other resources

In the past decades, several databases were built to investigate *Leishmania*; however, they most unfortunately often offline and no longer updated. LeishCyc (45) focused on biochemical pathways. LeishDB (46) included coding genes and non-RNAs and provided new annotation to them. The cysteine protease database in *Leishmania* species (47) was designed to find data related to cysteine protease and LeishBase (48) was a structural database. There are a few active databases: Leish-ExP (http://www.hpppi.iicb.res.in/Leish-ex) (which has not so far been published in a peer-reviewed journal) contains proteins exclusively present in *Leishmania*. Leish-ExP incorporates localization tools, includes GO annotations and calculates physico-chemical properties of proteins. LmSmdB (49) focuses on metabolic and biosynthetic pathways. TriTrypDB (20) is a kinetoplastid database that is part of the VEuPathDB resource (21). These databases contain a lot of experimental data and various tools to analyze eukaryotic pathogens, but they are mostly focused on genomic data—although proteomic datasets, and some protein prediction algorithms are also incorporated.

There are also a handful of databases that include information on host**–**pathogen interactions: HPIDB (50), PHIDIAS (51) and PHI-base (52) contain information about PPIs between the host and pathogen, while ImitateDB (53) specifically focuses on motif mimicry. These resources contain no or very little data about *Leishmania*.

In LeishMANIAdb, our main goal was to include protein information relevant to the infection and to complement previously established and still available resources. We included several proteomic datasets and enriched experimental information with state-of-the-art prediction tools. Still, the most powerful way to explore uncharted proteomes is to inspect MSAs and check for conserved residues and regions—LeishMANIAdb contains precalculated alignments for all proteins. We also added hundreds of annotations to thousands of proteins, including localization and interaction information. While several databases seem to be shut down after a couple of years, our laboratory hosts several resources and we routinely update them. We plan to do so with LeishMANIAdb as well as to expand its repertoire to host–*Leishmania* interactions involving glycans and glycolipids, which play major roles in the infection.

## Methods

### Resources

Protein sequences were retrieved from UniProtKB (release 2022_05) (22) and from TriTrypDB, release_59 (20) based on the UniProt cross-references (*L. brazliensis, L. donovani, L. infantum, L. major, L. mexicana, Bodo saltans, Leptomonas seymouri, Trypanosoma brucei, Trypanosoma cruzi, Trypanosoma rangeli* and *Trypanosoma theileri*). Homologs in other kinetoplastids and in SwissProt were searched with BLAST using E-value: <10^−5^; sequence identity >20%; and coverage >50%. In the ‘Homologs’ section, all results are displayed, and this information was used to calculate the “outgroup score” component of the motif score (see below and in Supplementary Material). In most other sections (and calculation), we only used homologous proteins until the first non-kinetoplastid SwissProt hit considering sequence identity (termed as ‘close homologs’, Supplementary Figure S1). Further similar kinetoplastid proteins were therefore considered as a different homology group, and this way huge superfamilies with a common ancestor from other species are split into smaller families. This consideration seemed to be useful for calculating expanded proteins. Furthermore, we downloaded strains belonging to the five selected *Leishmania* species from TriTrypDB. In this case, a more stringent condition was used in BLAST, by setting *E*-value: 10^-5^; sequence identity > 80%; coverage > 80%. All kinetoplastid species and strains were used to calculate motif conservation.

We prepared three different types of MSAs using ClustalΩ (54): (1) ‘non-redundant’ MSA using close homologous proteins from kinetoplastid reference proteomes; (2) the same MSA but with gaps removed from the ‘reference’ protein that is currently displayed on the webpage; and (3) a more redundant MSA using close homologous kinetoplastid proteins in all species and strains (used to calculate motif conservation).

High-throughput experiments were first mapped to the corresponding protein using the identifier provided in the original paper, then mirrored to close *Leishmania* close homologs if their sequence were identical.

IDRs were predicted using IUPred3 with default settings (long) (35) and using the AF2 models’ pLDDT and accessibility values—the latter was calculated by DSSP 3.1.5 with default settings (32), normalized using maximum values calculated as in Tien *et al.* (55), the exposed value threshold defined as suggested by Rost *et al.* (56). In the case of TM proteins, IDRs were also predicted by MemDis 1.0, sensitive settings (39). In this in-house modified version, the Position-Specific Scoring Matrices) were generated using kinetoplastid sequence library insead of SwissProt sequences. Furthermore secondary structure and accessibility were calculated using AlphaFold2 instead of sequence based predictors trained on distant proteins that was used in the original version. TM protein topology was predicted by CCTOP (34), however to minimize sporadic erroneous predictions, after an initial prediction we performed a constrained iteration where the topologies of close homologous proteins were used as a constraint. Using this approach, closely related proteins will likely have the same topology. Secondary structure elements derived from AF2 structures are also displayed. Pfam domains were identified using InterPro (33). Protein localization was displayed from the following tools: GO (41), DeepLoc (57) and SignalP6.0 (58). NetGPI (59) was used to predict GPI-anchors (all prediction results are displayed, therefore in case of a contradiction it is up to the user to evaluate the results). For these predictions, we used default settings and thresholds.

To detect SLiMs that may alter or rewire host cell regulation, we used the regular expressions from ELM (40) on all *Leishmania* sequences. We defined different contextual filters and merged them into a single score to rank motifs (for more details, see Supplementary Material): (1) Disordered: The score is the average of the IUPred3, AF2-based pLDDT and accessibility values. These disordered scores were first transformed so they range from 0 to 1, with 0.5 being the threshold, before calculating their mean; (2) Conservation of the motif was checked among close homologs with some permission for slight misalignment, and penalizing motifs that are present across all kinetoplastids—notably, in this case proteins from different *Leishmania* strains were also considered; (3) Localization: we used a simplified (intracellular/extracellular) distinction. Motif localization was determined using ELM GO annotations, secretion information and CCTOP prediction, while the binding domain localization was determined from TOPDOM (60). We looked for motif-domain pairs where they both have the same simplified (in/out) localization; (4) mRNA level: using transcriptomic experiments about expression data; (5) protein level: from experiments about protein abundance; (6) Secretion score based on secretome experiments; (7) Expansion score: reflecting how much the protein is expanded in *Leishmania* species (strains not included) compared to all kinetoplastids; (8) Outgroups score favoring proteins without homologs in SwissProt.

Structure data reflects structure data deposited in the PDB (61) before 26.03.2023, AlphaFold database (v3) and the TmAlphaFold database (v1). To generate Figure 3A we normalized the following graph: https://tmalphafold.ttk.hu/stati stics?parameters=MSZjMzllMDA0ME5EQXlNREU1TVRJ ek1DWXlNVFEzTkRnek5qVTJKakkwTVRVNU1Ua3hNRGdtTXpJM05qZz0%3D. All other data was downloaded in October, 2022 from the source databases.

### Manual curation

We manually curated hundreds of proteins, using two strategies. First, we searched PubMed and Google scholar for ‘*Leishmania* host–PPI and manually processed the results. Each protein in the experiments was mapped to the corresponding UniProt entry. Then, we mapped interaction data to the five *Leishmania* proteomes. When the experiment was performed on a protein from a different species, we mirrored it to the closest homology group in LeishMANIAdb, and we also indicated on the webpage that the experiment is from a different protein. All interactions were reported according to the community standard MIMIx level (23).

For annotating function/localization, we used the BLAST and alignment results from SwissProt (release 2022_05) (22) and kinetoplastid species, and information on annotated SwissProt entries if they were found. We also used publications when they were available (references are added to the website and to the downloaded files). We also used high-throughput studies on surface proteins (62, 63), and if a homologous protein was measured on the surface we took into account this information. We used prediction/annotation tools (Gene Ontology, DeepLoc, SignalP and NetGPI). These annotations were not made on proteins one by one, but rather for larger sets of kinetoplastid proteins belonging to the same family. We manually processed the entries using this approach, taking distant homologues, domain architectures and conservation patterns into consideration.

### Website design

The LeishMANIAdb website is written in PHP (v8.0) using the Laravel (v9.19) framework. All downloaded, predicted, or calculated data are stored in a local MySQL (v8.0) database. To visualize sequence features over amino acid sequences, we developed a javascript package using React (18.2), while 3D structures are visualized using the original (for non-TM proteins) or a locally modified version of Mol* (64) for TM proteins that can display the membrane bilayer (the modified version is available at: https://git.enzim.ttk.hu/web/TmMolStar). The modified version can display the membrane as two planes around the investigated TM protein using the results of TMDET 2.0 (65).

## Supplementary Material

baad074_Supp
