# Correction to: LeishMANIAdb: a comparative resource for *Leishmania* proteins

**DOI:** 10.1093/database/baae005

**Published:** 2024-01-16

This is a correction to: Gábor E Tusnády, András Zeke, Zsófia E Kálmán, Marie Fatoux, Sylvie Ricard-Blum, Toby J Gibson, Laszlo Dobson, LeishMANIAdb: a comparative resource for *Leishmania* proteins, *Database*, Volume 2023, https://doi.org/10.1093/database/baad074

In the originally published version of this manuscript there was a typographical error in the email for the corresponding author. This should read: dobson.laszlo@ttk.hu.

This error has been emended in the article.

