# Supplementary material for: LeishMANIAdb: a comparative resource for Leishmania proteins
Source: Database (Oxford). 2023 Oct 31;2023:baad074. doi: 10.1093/database/baad074 (PMC10627299; doi:10.1093/database/baad074)
Supplement: baad074_Supp [file baad074_supp.zip › SupplementaryMaterial_R2.docx]

Supplementary Material for

LeishMANIAdb: a comparative resource for *Leishmania* proteins

Gábor E. Tusnády^1,2^, András Zeke^1^, Zsófia E. Kálmán^3^, Marie Fatoux^4,5^, Sylvie Ricard-Blum^4^, Toby J. Gibson^6^, Laszlo Dobson^1,6^

^1^Protein Bioinformatics Research Group, Institute of Enzymology, Research Centre for Natural Sciences, Magyar Tudósok körútja 2, Budapest, 1117, Hungary

^2^Department of Bioinformatics, Semmelweis University, Tűzoltó u. 7, Budapest, 1094,

Hungary

^3^Faculty of Information Technology and Bionics, Pázmány Péter Catholic University, Práter u. 50/A, Budapest, 1083 Hungary

^4^ICBMS UMR CNRS 5246, University Lyon 1, Rue Victor Grignard, Villeurbanne, Lyon, 69622, France

^5^The work was done in UMR CNRS 5086, University Lyon 1, 7 Passage du Vercors, Lyon, 69367, France

^6^Structural and Computational Biology Unit, European Molecular Biology Laboratory, Meyerhofstraße 1, Heidelberg, 69117, Germany

**Supplementary Table 1**: *Leishmania* species in TriTrypDB and UniProtKB

|  | **Strains in TriTrypDB** | **Strain in UniProt** | **Crossreference from UniProt to TriTrypDB** | **UniProt proteome size** | **TriTrypDB proteome size** | **Sequence conflict** | **selected** |
| --- | --- | --- | --- | --- | --- | --- | --- |
| ***Leishmania major (MHOM/IL/81/Friedlin)*** | **4** | **1** | **yes** | **8038** | **8424** | **1615** | **yes** |
| ***Leishmania braziliensis (MHOM/BR/75/M2904)*** | **2** | **1 (duplicated proteome)** | **yes** | **8084** | **8176** | **100** | **yes** |
| ***Leishmania mexicana (strain MHOM/GT/2001/U1103) (MHOM/GT/2001/U1103)*** | **2** | **1** | **yes** | **8044** | **8144** | **51** | **yes** |
| ***Leishmania infantum (JPCM5)*** | **2** | **2** | **yes** | **8045** | **8527** | **913** | **yes** |
| ***Leishmania martiniquensis*** | ***2*** | ***1*** | ***no*** | ***7805*** | ***8482*** |  | **no** |
| ***Leishmania enriettii*** | ***2*** | ***1*** | ***no*** | ***8172*** | ***8730*** |  | **no** |
| ***Leishmania orientalis*** | ***1*** | ***1*** | ***no*** | ***8051*** | ***8158*** |  | **no** |
| ***Leishmania sp. Ghana 2012 LV757 (GH5)*** | ***0*** | ***1*** | ***no*** | ***8172*** | ***8119*** |  | **no** |
| ***Leishmania sp. Namibia (253)*** | ***0*** | ***1*** | ***no*** | ***8149*** | ***8266*** |  | **no** |
| ***Leishmania donovani (BPK282A1)*** | **6** | **6** | **yes** | **7960** | **7969** | **59** | **no** |
| ***Leishmania donovani (LdCL)*** | **6** | **6** | **yes** | **8326** | **8362** | **0** | **yes** |
| ***Leishmania donovani (FDAARGOS_361)*** | ***6*** | ***6*** | ***yes*** | ***7554*** | ***?*** |  | **no** |
| ***Leishmania donovani (FDAARGOS_360)*** | ***6*** | ***6*** | ***yes*** | ***7661*** | ***?*** |  | **no** |
| ***Leishmania donovani*** | ***6*** | ***6*** | ***yes*** | ***8165*** | ***?*** |  | **no** |
| ***Leishmania infantum*** | ***2*** | ***2*** | ***yes*** | ***8271*** | ***?*** |  | **no** |
| ***Leishmania tarentolae (Sauroleishmania tarentolae) (Parrot Tar II)*** | **1** | **1** | **yes** | **8387** | **8703** | **121** | **no** |

**
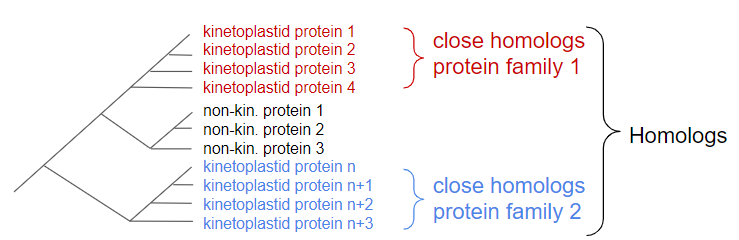
**

**Supplementary Figure 1**: Definition of homologs and close homolog in kinetoplastids.

**Scoring motifs**

Residue-based scores

1. Disordered score

Disordered score was calculated using the following formula

$$Motif_{disordered_{score}}=\frac{\sum_{i=start}^{end} {method\_score}_{i}}{n(end-start)}$$

Where $n=number of methods$; *start, end* marks the starting and ending position in the motif, respectively. In most cases three methods were incorporated (sometimes AlphaFold2 structure was missing).

$$IUPred=output probability of IUPred for a given position$$

$$AlphaFold2_{acc}=\left\{ \begin{aligned} \frac{RSA}{0.36*2} , ifRSA>0.36 \\ 0.5+\frac{\left( RSA+0.36 \right)}{(1-0.36)*2}, else \end{aligned} \right.$$

Where Relative Surface Accessibility (RSA) was calculated by normalizing DSSP accessibility output using values determined by [Tien et al.](about:blank) The 0.36 threshold for exposed residues was determined by [Rost et al.](about:blank) The equation yields 0.5 score at 0.36 value, and intermediate values between 0-0.36 and 0.36-1 are evenly rescaled.

$${AlphaFold2}_{pLDDT}=\left\{ \begin{aligned} \frac{\left( 1-\frac{pLDDT}{100} \right)}{\left( 1-0.7 \right)*2}, if pLDDT>70 \\ 0.5+\frac{\left( 1-\frac{pLDDT}{100} \right)-(1-0.7)}{0.7*2}, else \end{aligned} \right.$$

Where pLDDT is the confidence value for a given residue from AlphaFold2. The equation yields 0.5 score at 70 value, and intermediate values between 0-70 and 70 -100 are evenly rescaled. Note, that pLDDT inversely correlates with protein disorder.

Furthermore, any region listed in DisProt automatically gets maximum score (1). Transmembrane regions, signal peptides and PFAM domains get minimum score (0).

1. Conservation score

Conservation score was calculated on aligned Short Linear Motif pairs, when their distance were below 20 residues (permitting alignment errors)

$$\frac{\sum_{i=aligned\_motifs\_leishmania} 1-\frac{{distance}_{i}}{20}}{\sum_{i=aligned\_motifs\_trypanosomas} 1-\frac{{distance}_{i}}{20}}$$

Where ${distance}_{i}$ is the distance between two aligned motifs. The score is the highest when a motif is present in Leishmania, but missing from other Trypanosomas.

1. *Localization score

Localization score can be only calculated when there is evidence for both the motif and the corresponding domain localization. Currently there is a two-stage version was applied, where motifs and domains can be intracellular and extracellular.

Motif localization was determined using [ELM](about:blank) GO annotations, Protein secretion information (high-throughput experiments and SignalP), and [CCTOP](about:blank) prediction (in case of contradicting information this order was followed). Domain localization was determined using [TOPDOM](about:blank).

If the localization on the domain and motif side are the same, the score is 1, otherwise 0. (if there is not enough information this metric was not taken into account)

Protein scores:

1. *Maximum normalized mRNA expression score (using experiments by [Lahav et al](about:blank).)
2. *Maximum normalized protein expression score (using experiments by [Lahav et al](about:blank).)
3. $Secretion score=\frac{\sum_{i=sets\_for\_species} x_{i}}{n}$

Where n is the number of sets covering the species and

$$\left\{ \begin{aligned} x_{i}=1, if included in the secretion set \\ x_{i}=0, if not included in the secretion set \end{aligned} \right.$$

1. $Expansion score=\frac{\sum_{i=leishmania\_species} x_{i}}{\sum_{i=trypanosoma species} x_{i}}$

Where

$$\left\{ \begin{aligned} x_{i}=1, if protein present in the species \\ x_{i}=0, else \end{aligned} \right.$$

1. Outgroup score: Penalizing proteins with homologs in non-Trypanosomes. For this step all proteins were searched against SwissProt using BLAST.

$$outgroup\_score=1-highest\_sequence\_identity$$

Scores with * cannot be applied for all proteins or motifs. The weight of different scores is depending on the scores that can be calculated (see tables below).

Residue-based scores (total weight 0.7)

|  | With localization score | No localizations score |
| --- | --- | --- |
| Disordered score | 0.25 | 0.35 |
| Conservation score | 0.25 | 0.35 |
| Localization score | 0.2 | 0 |

Protein scores (total weight: 0.3) :

|  | With expression scores | No expression scores |
| --- | --- | --- |
| mRNA expression score | 0.06 | 0 |
| Protein expression score | 0.06 | 0 |
| Secretion score | 0.06 | 0.1 |
| Expansion score | 0.06 | 0.1 |
| Closest homolog | 0.06 | 0.1 |
